# A Case of Erythema Multiforme Following the Administration of Nemolizumab

**DOI:** 10.7759/cureus.79070

**Published:** 2025-02-15

**Authors:** Yoshihito Mima, Tsutomu Ohtsuka

**Affiliations:** 1 Department of Dermatology, Tokyo Metropolitan Police Hospital, Tokyo, JPN; 2 Department of Dermatology, International University of Health and Welfare Hospital, Tochigi, JPN

**Keywords:** erythema multiforme, interleukin-31, nemolizumab, prurigo nodularis, t helper 1, t helper 2

## Abstract

Prurigo nodularis (PN) is a chronic inflammatory skin disorder characterized by intensely pruritic nodules. The pathogenesis of PN involves immune dysregulation, with a predominance of T helper (Th2)-type inflammation, including interleukin (IL)-4, IL-5, IL-13, and IL-31. Nemolizumab, an IL-31 receptor A inhibitor, was newly approved for PN in 2024. We report a case of erythema multiforme (EM) that developed three weeks after the first administration of nemolizumab. Three weeks post-injection, she developed multiple targetoid edematous erythematous patches and plaques on her trunk and limbs, leading to a clinical diagnosis of EM. Since she had no new medications and no recent history of infections, we inferred that the development of EM was likely associated with the administration of nemolizumab. The pathophysiology of EM involves a predominant Th1-driven inflammatory response. Therefore, nemolizumab likely suppressed Th2 inflammation, leading to compensatory Th1 hyperactivation, which may have triggered EM. To our knowledge, this may be the first reported case of EM following nemolizumab treatment for PN. Further research is necessary to investigate its immunomodulatory effects and potential adverse events.

## Introduction

Prurigo nodularis (PN) is a chronic inflammatory skin disorder characterized by intensely pruritic papules, pustules, and nodules. These nodules may appear flesh-colored, erythematous, or hyperpigmented and typically present in a symmetrical linear distribution on the extensor surfaces of the limbs and trunk [1]. The condition worsens with repeated scratching, leading to chronic lesions and disease progression [1]. Several risk factors of PN development include psychiatric disorders, malignancies, liver failure, chronic kidney disease, diabetes, and chronic infections such as human immunodeficiency virus [2].

The pathogenesis of PN is thought to be driven by a chronic pruritus-scratch cycle, a distinct skin reaction pattern triggered by persistent itching and scratching. Although the precise etiology remains unclear, dysregulation of the immune and nervous systems plays a crucial role in perpetuating this cycle [3]. Lesional skin in PN exhibits infiltration by immune cells, such as eosinophils, neutrophils, macrophages, mast cells, and T cells, with a predominance of CD4-positive T helper (Th)2 cells, suggesting a Th2-skewed inflammatory response [4]. Additional mediators, such as tryptase, eosinophilic cationic protein, histamine, prostaglandins, and neuropeptides, are also implicated in the inflammatory process [5]. Neuroregulatory abnormalities contribute to PN pathogenesis as well. Alterations in dermal and epidermal nerve fiber density have been observed in PN lesions, with increased expression of neuropeptides such as calcitonin gene-related peptide and substance P [6].

Treatment options for PN encompass a broad spectrum, including topical therapies, such as corticosteroids and calcineurin inhibitors, phototherapy, systemic agents, such as methotrexate and cyclosporine, and oral anticonvulsants, antidepressants or antihistamines [2]. Topical treatments are the first-line option, and if they are ineffective, phototherapy or oral antihistamines for symptomatic relief of pruritus are considered. If these treatments fail to provide sufficient improvement, more intensive therapies, such as methotrexate or cyclosporine, may be considered in consultation with the patient [2]. Additionally, nemolizumab, a once-monthly anti-interleukin (IL)-31 receptor A monoclonal antibody, was approved in 2024 in Japan as a novel treatment for PN [7]. In a phase III randomized, double-blind, placebo-controlled trial, nemolizumab demonstrated significant improvements in the numeric rating scale (NRS) for pruritus at week 16, with a higher proportion of patients achieving an NRS reduction of ≥4 and PN-IGA (Investigator’s Global Assessment) scores of 0 or 1 [8]. Additionally, nemolizumab exhibited a rapid onset of action, with significant reductions in pruritus scores and sleep disturbances within days of administration [9]. However, adverse effects of nemolizumab have been reported, including exacerbation of preexisting eczematous lesions, edematous erythema, bullous pemphigoid (BP), and psoriasiform dermatitis [10-13].

Erythema multiforme is characterized by annular, erythematous target-like lesions that can progressively spread across the body over time. In some cases, vesicles may form within the erythematous plaques, and mucosal involvement of the eyes and mouth, as well as systemic symptoms, such as fever, may also occur. The condition is believed to result from an immune-mediated response triggered by infections or drug exposure. Historically, herpes simplex virus (HSV) and Mycoplasma infections have been the most common infectious causes of erythema multiforme. However, in recent years, there has been an increasing number of reports linking COVID-19 to erythema multiforme [14-16]. Drug-induced erythema multiforme has been associated with a wide range of medications, including antibiotics, antipyretic analgesics, anticonvulsants, contrast agents, and anticancer drugs. In drug-induced cases, skin lesions typically appear days to weeks after drug administration. EM requires differentiation from Stevens-Johnson syndrome (SJS) and toxic epidermal necrolysis (TEN). If the causative drug is not discontinued and continues to be administered indiscriminately, there is a risk of progression to SJS or TEN, necessitating careful attention.

Herein, we report a case of erythema multiforme (EM) that developed three weeks after the administration of nemolizumab for PN.

## Case presentation

**Figure 1:**
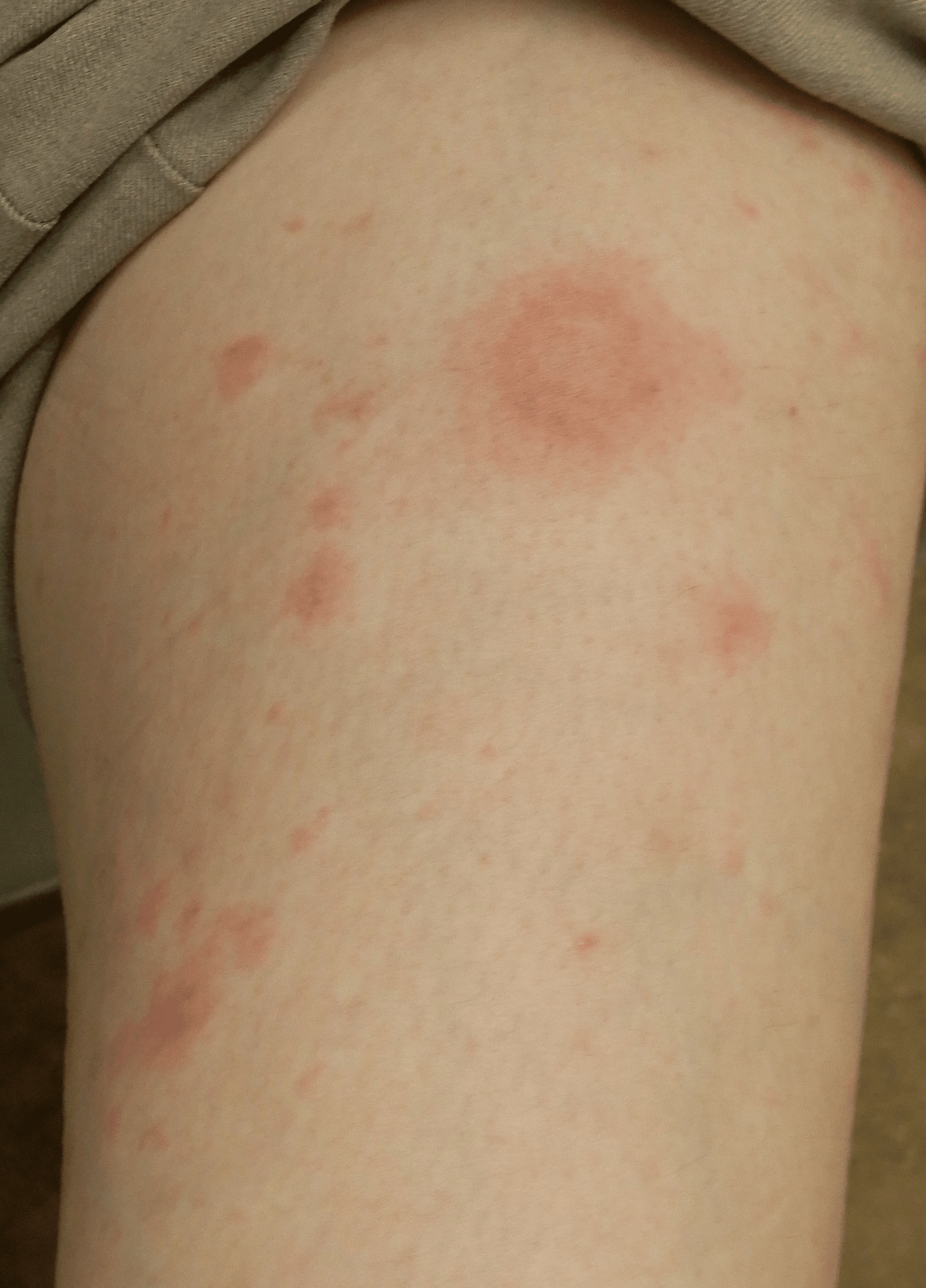
Multiple targetoid edematous erythematous plaques on her lower legs

**Figure 2:**
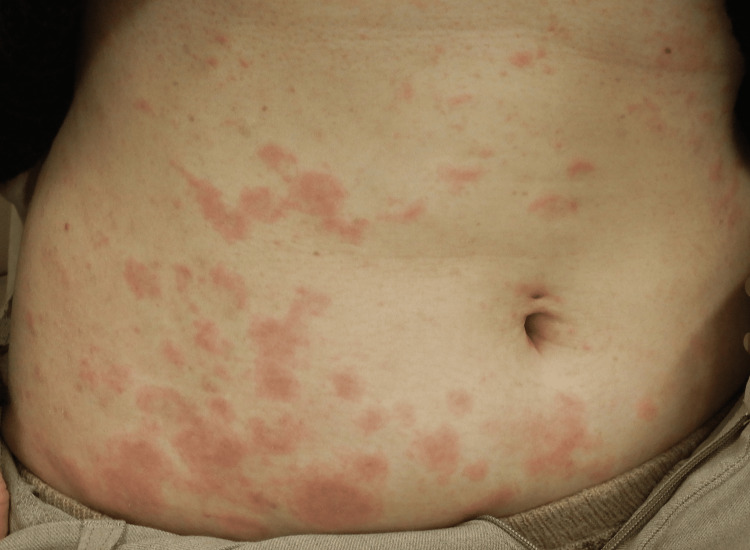
Multiple targetoid edematous erythematous plaques on her trunk

A 46-year-old woman with no significant medical history and no regular use of medications or supplements presented with persistent pruritic nodules. Two years prior, she developed insect bites and eczema on both lower legs. While the eczema resolved with topical corticosteroids and oral histamines, persistent pruritus led to repeated scratching, resulting in the formation of prurigo nodules on both lower extremities. Despite intensified topical corticosteroid therapy and adjustments in antihistamine treatment, her condition remained refractory for over a year, prompting referral to a dermatology clinic for further management (June 2024). Physical examination revealed multiple brownish skin nodules and excoriation marks bilaterally on the lower legs, with no systemic symptoms. Based on these clinical findings, a diagnosis of nodular prurigo secondary to persistent scratching following insect bites was made. As the existing topical corticosteroid and antihistamine therapies provided insufficient relief, and her pruritus remained severe, systemic corticosteroids were introduced. However, pruritus flared upon tapering the corticosteroid dosage, with a consistently high NRS score of 9-10, severely impacting her daily life and sleep. Given the refractory nature of her symptoms, treatment with nemolizumab, newly approved in 2024, was initiated (November 2024). Following the administration of nemolizumab, her pruritus improved dramatically, with an NRS score of 0 within one month, even without oral antihistamines. However, three weeks after the first dose, multiple targetoid edematous erythematous plaques, ranging from the size of a thenar eminence to a clenched fist, were scattered across the trunk and limbs (Figures 1, 2).

The eruptions were not accompanied by pruritus or pain. Based on these characteristic clinical findings, EM was diagnosed. She had no history of influenza or COVID-19 vaccination in the past year. Serologic tests for herpes simplex virus immunoglobulin (Ig) M and Mycoplasma antibodies were negative, and no abnormal findings were observed in the laboratory examination (Table 1). Additionally, there were no signs of infection such as fever, malaise, vesicles, or pustules. Her only oral medication was olopatadine at the time of its appearance, which had remained unchanged for the past three months. Given these findings, nemolizumab was considered a potential trigger for the development of EM. Although a skin biopsy of the lesion was suggested, the patient declined due to the absence of pruritus. Drug hypersensitivity testing, including the drug-induced lymphocyte stimulation test and patch testing, was considered but could not be performed due to the unavailability of nemolizumab's raw formulation. Treatment was modified to include topical clobetasol propionate ointment and a short course of oral corticosteroid therapy (10 mg/day), leading to the complete resolution of the rash. As the antipruritic effect of nemolizumab persisted, maintaining an NRS score of 0, further administration was deemed unnecessary, and the treatment was discontinued after a single dose. The patient has since continued treatment with betamethasone valerate ointment and oral olopatadine.

**Table 1:** Results of the laboratory examination at the emergence of erythema multiforme RR = Reference range; AST = Aspartate aminotransferase; ALT = Alanine aminotransferase; LDH = Lactate dehydrogenase; CRP = C-reactive protein; WBC = White blood cell; HSV = Herpes simplex virus; IgM = Immunoglobulin M

Variable	Patient value	RR, adults
AST	18 U/L	11–33 U/L
ALT	12 U/L	6–37 U/L
LDH	142 U/L	135–214 U/L
CRP	0.10 mg/dL	< 0.30 mg/dL
WBC	4,700 /μL	3,500～9,000 /μL
Eosinophil	450 /μL	30～500 /μL
HSV IgM	Nonreactive	Nonreactive
Mycoplasma pneumoniae antibody titer	Nonreactive	Nonreactive

## Discussion

From an immunological perspective, an increase in Th2-associated cytokines, including IL-4, IL-5, IL-10, IL-13, and IL-31, has been reported in the dermis of PN. Among these, IL-31 is particularly significant, as it not only induces neuroinflammation and triggers pruritus but also acts on fibroblasts to promote fibrosis. Therefore, IL-31 is considered a key factor in both pruritus induction and the formation of nodules in PN. Given its crucial role, inhibiting IL-31 is regarded as highly important in the treatment of PN, making IL-31 inhibitors a promising therapeutic option for this condition [7-9].

On the other hand, in EM, infection or drug exposure leads to the excessive expression of inflammatory mediators, such as interferon-gamma (IFN-γ), tumor necrosis factor-alpha (TNF-α), perforin, and granzyme B, resulting in severe skin inflammation, erythema, and epidermal destruction [15,16]. Lesional skin in EM exhibits a predominantly Th1-driven inflammatory response, with IFN-γ and TNF-α playing key roles in disease pathogenesis. These inflammatory mediators are thought to be released via autoreactive T cells, ultimately driving the formation of erythematous lesions [15-17]. Therefore, EM is primarily considered a Th1-mediated inflammatory condition [15-17].

T-cell immunity can be broadly classified into two major pathways: Th1 inflammation and Th2 inflammation [18]. Th1 inflammation primarily defends against intracellular pathogens, such as viruses and bacteria, by activating cell-mediated immunity to eliminate infected cells. It is regulated by pro-inflammatory cytokines, including IFN-γ, TNF-α, and IL-2. Th2 inflammation is closely associated with allergic reactions and protects against extracellular pathogens by promoting antibody production. Key cytokines involved in Th2 inflammation include IL-4, IL-5, IL-13, and IL-31. Th1 and Th2 pathways exist in a reciprocal balance, where the activation of one pathway generally suppresses the other, maintaining immune homeostasis [18]. In addition to Th1 and Th2, Th17 and regulatory T cells (Tregs) are also crucial components of T-cell immunity. These four inflammatory pathways interact dynamically, counterbalancing each other to regulate immune responses. In cutaneous inflammatory diseases, dysregulation of this balance - either through excessive activation or suppression of one pathway - can disrupt immune homeostasis, leading to pathological inflammation and disease progression [19]. A well-known example is the use of dupilumab, which inhibits the Th2 cytokines, IL-4 and IL-13. This can lead to compensatory hyperactivation of Th1 inflammation, potentially triggering alopecia areata, or Th17 inflammation, which may induce psoriasiform dermatitis [20]. Similarly, IL-31 inhibitors, such as nemolizumab, have been associated with skin-related adverse effects due to immune dysregulation [10-13].

Nemolizumab, which was recently approved for PN in 2024, targets IL-31 to suppress Th2 inflammation [7]. However, this suppression may lead to compensatory increases in Th17-mediated inflammation, potentially triggering psoriasis-like eruptions [13]. Additionally, reports have linked nemolizumab to exacerbations of atopic dermatitis (AD), BP, and urticaria - conditions primarily driven by Th2 cytokines such as IL-4, IL-5, and IL-13. Blocking IL-31, a Th2 cytokine, may paradoxically lead to increased production of these Th2 cytokines, thereby intensifying type 2 inflammation [10-12].

The skin-related adverse effects of nemolizumab may be attributed to immune imbalance [10-13,20]. In this case report, we present a patient who developed EM following the initial administration of nemolizumab. It is possible that IL-31 inhibition led to Th2 suppression, resulting in compensatory Th1 hyperactivation and subsequent EM occurrence. To date, no prior reports of EM associated with nemolizumab administration have been documented. However, the key limitation of this study is that we were unable to perform confirmatory allergy tests, such as patch testing or drug lymphocyte stimulation tests, as the manufacturer did not provide access to the drug, and we didn’t rechallenge with the administration of nemolizumab. Given the recent approval of nemolizumab for the treatment of PN, additional adverse events may emerge over time [7]. Accumulating data on these events is not only beneficial for patients receiving nemolizumab but also crucial for elucidating the immunological mechanisms underlying its adverse effects. Further investigation into the cytokine and immune response alterations induced by IL-31 inhibition may contribute to a deeper understanding of its role in the pathophysiology of cutaneous inflammatory diseases such as AD and PN. Continued case reports and research will be essential in clarifying the broader immunological impacts of IL-31 inhibition.

## Conclusions

Nemolizumab, an IL-31 receptor A inhibitor, was newly approved for nodular prurigo in 2024 in Japan. By blocking IL-31 signaling, nemolizumab may disrupt T cell immune balance, potentially triggering inflammatory skin disorders such as bullous pemphigoid, urticaria, and psoriasiform dermatitis. Herein, we report a case of erythema multiforme following nemolizumab administration. While no previous cases of erythema multiforme associated with nemolizumab have been reported, it is possible that suppression of Th2 inflammation due to nemolizumab led to compensatory Th1 hyperactivity, contributing to the development of erythema multiforme. Further case studies and research are needed to better understand the immune balance shifts induced by nemolizumab.
